# Supplementary material for: The interactome of intact mitochondria by cross-linking mass spectrometry provides evidence for coexisting respiratory supercomplexes
Source: Mol Cell Proteomics. 2017 Dec 8;17(2):216–32. doi: 10.1074/mcp.RA117.000470 (PMC5795388; doi:10.1074/mcp.RA117.000470)
Supplement: Supplemental Data [file supp_RA117.000470_133922_0_supp_23978_fzffwf.zip › spectra_annotation/mito_spectra_annotation/21-1-7-1-9-1.pdf]

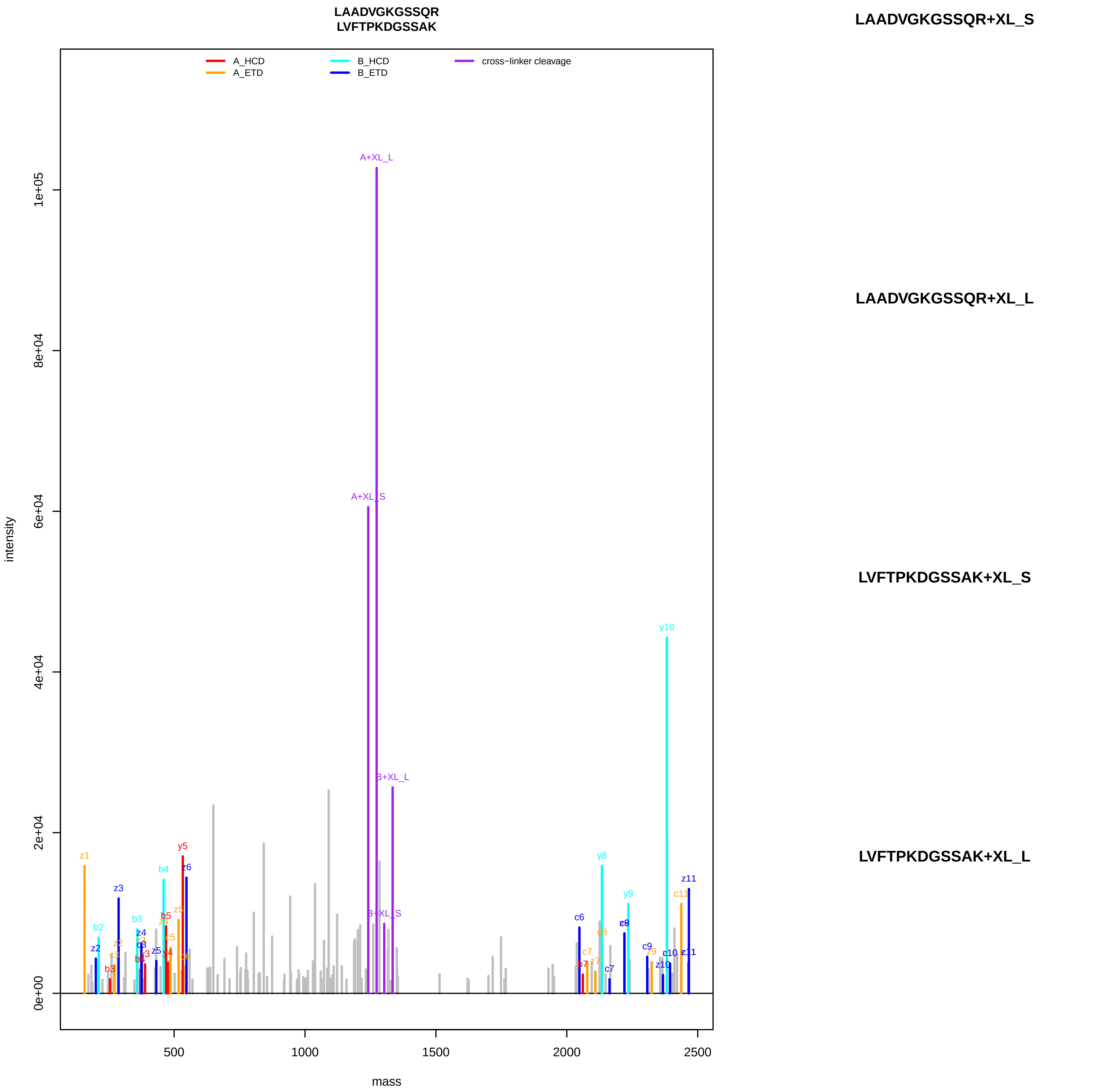
LAADVGKGSSQR
LVFTPKDGSSAK
A_HCD
A_ETD
B_HCD
B_ETD
cross−linker cleavage
A+XL_L
A+XL_S
B+XL_L
B+XL_S
y10
y8
y9
z11
c11
c6
c8
z8
c9
z9
c10
z10
c7
z7
b7
z1
b2
z2
z3
b3
c3
z4
b4
c2
z2
c4
z5
y5
z6
b5
z5
c5
y4
y3
b3
intensity
mass
0e+00
2e+04
4e+04
6e+04
8e+04
1e+05
500
1000
1500
2000
2500
LAADVGKGSSQR+XL_S
LAADVGKGSSQR+XL_L
LVFTPKDGSSAK+XL_S
LVFTPKDGSSAK+XL_L
